# Rural-urban disparities in healthcare factors and long-term health outcomes in individuals with pediatric-onset spinal cord injury

**DOI:** 10.3389/fresc.2023.1102441

**Published:** 2023-05-19

**Authors:** Kyle C. Deane, Nikhil Kurapati, Emma Gill, Lawrence C. Vogel, Kathy Zebracki

**Affiliations:** ^1^ Department of Psychology, Shriners Children's Chicago, Chicago, IL, United States; ^2^ Department of Psychology, Rosalind Franklin University of Medicine and Science, North Chicago, IL, United States; ^3^ Department of Family Medicine, Soin Medical Center, Beavercreek, OH, United States; ^4^ Department of Psychiatry, Northwell Health, Queens, NY, United States; ^5^ Department of Pediatrics, Rush Medical College, Chicago, IL, United States; ^6^ Department of Psychiatry and Behavioral Sciences, Northwestern University Feinberg School of Medicine, Chicago, IL, United States

**Keywords:** pediatric, spinal cord injury, health disparities, urban, rural, health care access, long-term outcomes

## Abstract

**Objective:**

Adults with pediatric-onset spinal cord injury (SCI) require long-term care and demonstrate elevated risk of secondary health conditions and psychosocial challenges. Medical providers are typically found in more populous and wealthy areas, resulting in a relative lack of providers in rural areas, a discrepancy even more pronounced among specialty providers. As a result, those who reside in rural regions potentially have unmet medical needs, representing a significant public health concern. The purpose of this study was to assess differences between rural and urban-residing participants with pediatric-onset SCI in factors affecting healthcare usage (e.g., employment, income, access to private insurance, community integration) and long-term healthcare outcomes (i.e., secondary health conditions and psychosocial functioning).

**Methods:**

Data were gathered from an ongoing study examining long-term outcomes of adults with pediatric-onset SCI. Participants (*N* = 490) completed measures of sociodemographics, injury characteristics, and medical outcomes. Participant zip codes were classified as rural or urban using the ProximityOne database based on the ZIP Code Tabulation Areas from the 2020 census.

**Results:**

Individuals residing in rural regions report lower levels of education, income, employment rates, private health insurance, and community integration (mobility, occupation, and social engagement), as well increased incidence of pressure injuries, urinary tract infections, hospitalizations, bowel incontinence, sleep difficulties, and perceived physical health. No differences in incidence of psychosocial functioning were identified.

**Conclusion:**

Mitigating identified disparities and obstacles to treatment of SCI due to residing in rural environments would result in important improvements in treatment outcomes and future prevention efforts of secondary health complications, improving the overall health of adults with pediatric-onset SCI.

## 1. Introduction

Accessing timely and comprehensive healthcare can have significant effects on the promotion and maintenance of long-term health in individuals with chronic medical conditions, such as spinal cord injuries (SCIs). SCIs are typically sudden and debilitating injuries that result in long-term motor, sensory, and autonomic dysfunction, as well as altered community participation and quality of life (1). Individuals with SCI often require long-term care for secondary health conditions including urinary tract disorders, as pressure injuries, neurogenic bowel and bladder, pain, and sleep challenges (2, 3). Those with SCI may also experience higher rates of hospitalization related to these secondary health complications following their initial injury (4). Hospitalizations can further lead to reduced job acquisition and retention in adults with SCI (5), negatively affecting financial outcomes and quality of life.

In addition to secondary health conditions, individuals with SCI have an increased risk of experiencing psychological morbidity. A systematic review found that nearly 30% of individuals with SCI have elevated depressive symptoms (6), a significantly higher rate of than found in the general medical population (7). Similar estimates have been found for the prevalence of depression in adults with pediatric-onset SCI (8). Craig, Tran, and Middleton (6) also demonstrated that individuals with SCI are at risk for increased anxiety. Moreover, there are identified associations between occurrence of pediatric-onset SCI and reduced satisfaction with life (9) and negative subjective ratings of health (10).

Relatively few studies have focused on access to adequate health and support services for individuals living with SCI in rural regions. Individuals with SCI living in rural environments may encounter multiple barriers in accessing care vs. their urban-living counterparts, including timely access to primary and specialized healthcare services and transportation challenges (11). These barriers may result in delayed diagnosis and treatment of preventable conditions (2). Reduced access to transportation to healthcare is predictive of increased secondary health conditions in persons with high-level SCI (12). There is a lack of healthcare practitioners sufficiently skilled in care for individuals with SCI given the relative complexity and infrequency of the condition. Additionally, any shortages of advanced diagnostic and treatment equipment may be more pronounced in rural regions (2). Access to personal means of transportation, inadequate public transportation, and physical and architectural barriers such as lack of accessibility ramps and automated doors have also been cited as preventing healthcare utilization (13).

SCI as well as its associated health conditions appears to also influence community engagement, employment, and occupational functioning. In addition to SCI-specialized rehabilitative and healthcare resources, urban areas have more options for employment and recreation (14) and individuals with physical disabilities living in urban areas typically receive more vocational services (15). Paid employment and other types of participation have been linked to reduced rates of depression over time (3), higher life expectancy (16), higher social integration (17), and increased life satisfaction (3). Rural locations tend to lack opportunities to engage in health-promoting activities as well as complementary therapies, sports, peer support, equipment, and mobility-related services (2). In addition, financial burdens, such as lower incomes, less education, and lower quality health insurance, may also contribute to the poorer health outcomes in this population (18, 19).

Individuals who experience injury during childhood or adolescence, who account for approximately 20% of SCIs (20), represent a distinct subset of those with SCI and geographical region may influence outcomes differently for this group. Individuals with pediatric-onset may be more likely to have little work experience prior to the injury, may be more likely to be pursuing education at the time of the injury, and may be especially sensitive to community factors with respect to long-term health outcomes of SCI. In terms of educational attainment, individuals living with pediatric-onset SCI are more likely to pursue higher learning, with higher rates of college/associate degree attainment than the general population (41.1% vs. 28.3%). Vogel and colleagues (21) reported an employment rate of 42%–69% in this population, which is greater than the average rate of 19%–23% for those who sustained SCI as adults, potentially influencing healthcare access factors like income, health insurance, and out-of-pocket medical expenses. There may also be differences observed in psychosocial functioning. For example, some studies of adults with pediatric-onset SCI have revealed reduced incidence of depressive symptoms as compared with individuals with adult-onset SCI (10). It is therefore imperative to examine the role of rural living on the health and wellness of individuals with pediatric-onset SCI.

To the authors’ knowledge, prior studies have not examined how geographical location (rural vs. urban-living) affects the overall accessibility of care and health outcomes in individuals with pediatric-onset SCI. As noted, the health outcomes of individuals with pediatric-onset SCI living in rural vs. urban environments are widely unexamined. This is a problem given that individuals with pediatric-onset differ from adult-onset injuries in a number of ways (22). The identification of disparities in health resources in rural areas would help to improve the care of individuals with SCI residing in those areas. To this end, the current study seeks to examine variations in healthcare factors, secondary health conditions, and psychosocial outcomes in rural vs. urban-living individuals with pediatric-onset SCI. We predicted that rural-living participants would have elevated structural and healthcare challenges (i.e., increased healthcare expenses, lower quality health insurance, reduced employment, and lower community integration). We also hypothesized that rural-living individuals with pediatric-onset SCI would experience more secondary health conditions (pressure injuries, urinary tract infections, bladder/bowel incontinence, hospitalizations, pain, and sleep difficulties) and psychosocial health issues (i.e., anxiety, depressive symptoms, reduced satisfaction with life and happiness, and lower perceived mental and physical health) compared to those living in urban environments.

## 2. Materials and methods

Inclusion criteria included participants that were adult individuals who sustained an SCI at less than 19 years of age and were living in the United States. Exclusion criteria included individuals who were unable to communicate in English or who had a significant brain injury. Participants all received care at a pediatric SCI program (either Shriners Children’s Chicago, Philadelphia, and Northern California). These programs treat both rural and urban-residing patients from central, eastern, and western regions of the United States. There are no financial or insurance restrictions and care is provided free of charge at these SCI programs. Informed consent was obtained from all participants in the study, and participation involved completing annual phone interviews with trained research specialists over a period of nearly thirty years. As this study utilized a convenience sampling method, no randomization, power analysis, or blinding was performed. Approval from the Institutional Review Board *via* Western Institutional Review Board was obtained for this study, which is a part of a larger follow-up study on long-term outcomes of adults with pediatric-onset SCI.

Individuals aged 19–55 were interviewed using a study-specific structured questionnaire assessing sociodemographic characteristics, healthcare resources, secondary health conditions, and psychosocial functioning. Demographic and healthcare access data collected included race, education level, employment status, income, insurance, and medical expenses. Initial injury-related information was obtained from medical records and the Shriners Children’s SCI database. Injury severity (American Spinal Injury Association Impairment Scale; AIS) and level were established using the *International Standards for Neurological Classification of Spinal Cord Injury* (23).

**Figure 1:**
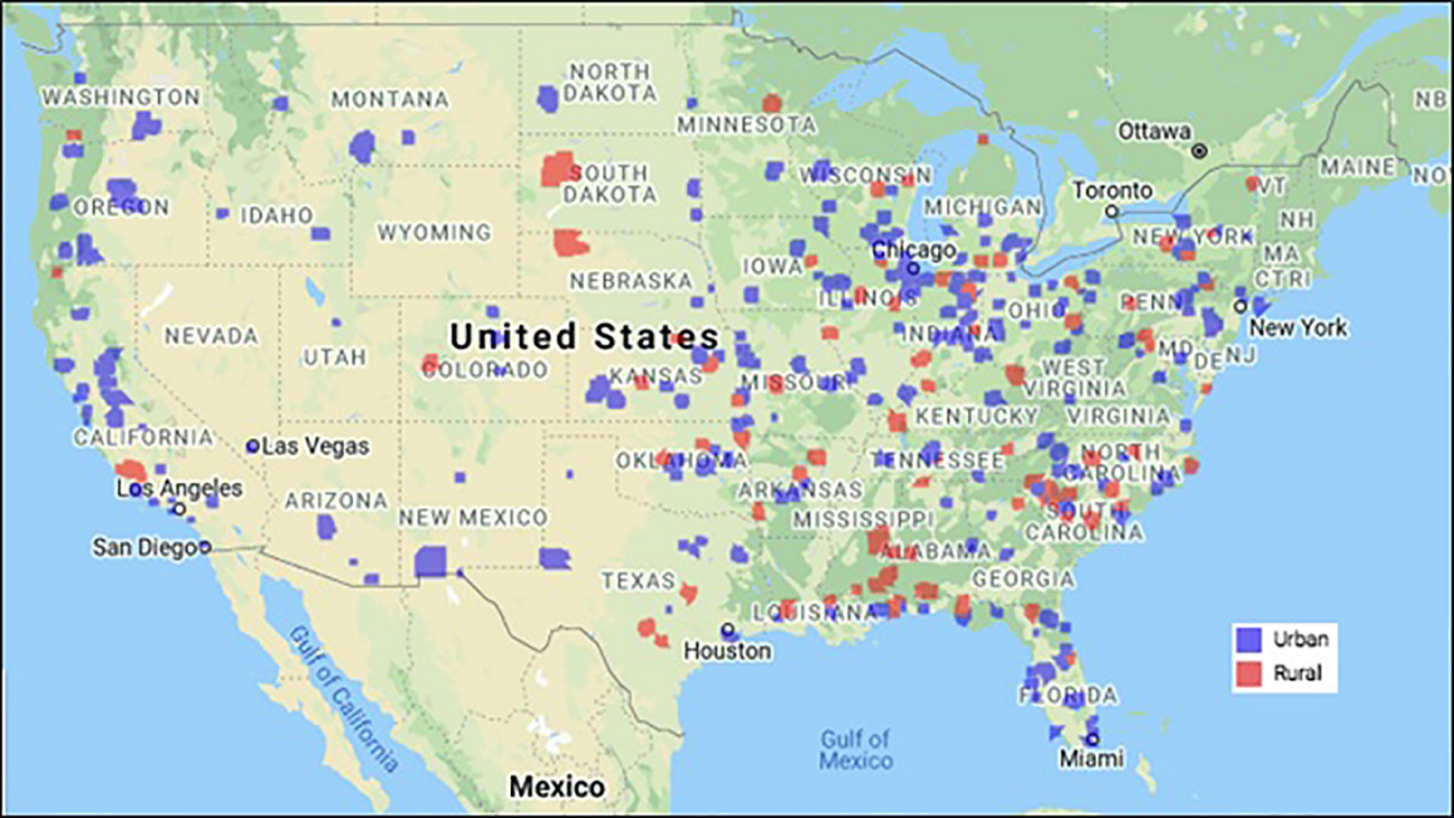
Map of urban and rural ZIP codes represented in the study.

To assign participants to the appropriate geographical subtype (urban vs. rural), participant zip codes corresponding to their most recent mailing address on file were entered into the ProximityOne database (24). ProximityOne is a program that can geocode rurality level based on zip code. The program uses data from the 2020 Census and relies on ZIP Code Tabulation Areas (ZCTAs), which provide information as to the geographic makeup of different ZIP code areas within the United States. The most recent zip code for each participant was entered into the database and was subsequently designated as either urban or rural by the program. Figure 1 presents a map depicting the rural and urban ZIP codes represented in the study.

Secondary health conditions were assessed through the structured study questionnaire self-report as well as a series of standardized self-report measures. The occurrence of secondary health conditions in the previous year was recorded and included pressure injuries, urinary tract infections (UTIs), bladder and bowel accidents, hospitalizations due to medical conditions. Pain was also assessed in terms of frequency using a six-point numerical rating scale (ranging from 1 “less than once per month” to 6 “daily”) and current intensity assessed using an 11-point scale (ranging from 0 “no pain” to 10 “very intense pain”). Subjective sleep quality was obtained with the Pittsburgh Sleep Quality Index (PSQI) (25). This measure contains queries related to sleep latency, interval, interruption, and restoration over the past month. The PSQI has been demonstrated to be a reliable and valid measure of sleep quality among individuals with SCI (26). The SF-12v2 Health Survey (27) was employed to examine self-perceived mental and physical health-related quality of life, with higher scores indicating higher levels of perceived health.

Standardized self-report measures were utilized to assess psychosocial outcomes and community integration. The Patient Health Questionnaire-9 (PHQ-9) (28) assesses depressive symptoms using diagnostic criteria for major depressive disorder according to the Diagnostic and Statistical Manual, Fourth Edition. The Beck Anxiety Inventory (BAI) was used to assess current symptoms of anxiety (29). The General Happiness Scale is a global measure of subjective happiness (30), and the Satisfaction with Life Scale is a measure of global life satisfaction (31). Community participation, integration, and activity level was obtained using the Craig Handicap Assessment and Recording Technique (CHART) (32). The CHART is categorized into 6 domains: Physical Independence, Cognitive Independence, Mobility, Occupation, Social Integration, and Economic Self- Sufficiency. Higher scores on the CHART represent higher community integration.

### 2.1. Analytic approach

Preliminary analyses were conducted to compute descriptive statistics of participant demographic and injury characteristics as well as to evaluate assumptions of normality. Subsequently, analyses were performed to examine whether injury and sociodemographic factors differed related to geographic location (urban vs. rural living). Mann–Whitney *U* was used to compare groups on non-normally distributed continuous variables (e.g., household income) and *t*-tests to examine normally distributed continuous variables (e.g., age). Pearson’s chi-squared analyses were used to compare groups on categorical variables (e.g., etiology). Lastly, to investigate if geographical location was related to secondary health conditions and psychosocial outcomes, a sequence of logistic regression analyses were conducted. In these models, the health and psychosocial outcome served as the dependent variable and rural living status served as a predictor variable, with age at injury, race (Caucasian vs. other ethnicity), and injury level (paraplegia vs. tetraplegia) serving as controls given the role of these variables in predicting health outcomes in other investigations (7, 33–35). Adjusted odds ratios (OR) with 95% confidence intervals (CI) were calculated. Analyses were carried out using IBM SPSS (IBM SPSS Statistics for Windows 27.0).

## 3. Results

Of the 497 total individuals in the broader study, 7 were omitted in the present study due to their most current address corresponding to a location outside of the United States. Of the remaining 490 participants included in the current study, 62.4% (*n *= 306) were biological male and 85.8% (*n *= 416) were Caucasian. The average age of individuals was 34.18 years (SD = 7.49; range, 19–55 years), average injury year was 14.19 (SD* *= 4.46; range, 0–18), and the mean length of injury was 20.01 years (SD* *= 8.51; range, 1–54). 53.7% (*n *= 262) had tetraplegia injuries and 68.8% (*n *= 326) of injuries were complete. Neurological classification of injury was as follows: C1–C4 AIS A, B, or C (*n *= 67; 13.9%); C5–C8 AIS A, B, or C (*n *= 164; 34.1%); T1-S5 AIS A, B, or C (*n *= 203; 42.2%); and AIS D (*n *= 47; 9.8%). In terms of etiology, individuals sustained their SCI as a result of vehicular or pedestrian collision (*n *= 244; 50.2%), sports (*n *= 118; 24.3%), medical or surgical complications (*n *= 50; 10.3%), violence (*n *= 31; 6.4%) and nonviolent falls (*n *= 31; 6.4%). Approximately half of individuals described being employed (*n *= 233; 47.6%), and most indicated they had not obtained a bachelor’s degree or higher (*n *= 280; 57.1%).

Table 1 presents the demographic and injury characteristics varying as a function geographic location (rural vs. urban). No significant differences between these groups in terms of age, sex, race, age at injury, injury duration, injury level or severity, or injury etiology was identified. Table 2 presents a comparison of these two groups in terms of various structural and healthcare characteristics. Compared with those living in urban environments, there was a greater proportion of individuals living in rural areas that were significantly less likely to have a college or professional degree (*p* < .001), more likely to be unemployed (*p* < .001), and increased likelihood of reporting having no health care insurance or public health insurance (*p* = .015). Additionally, rural-living individuals had significantly lower personal and household incomes (difference of approximately $19,000 on both measures), worked more hours per week on average, and required more hours of unpaid personal care assistance. These groups did not significantly differ in terms of paid hours of personal care assistance or out of pocket medical expenses.

**Table 1:** Comparison demographic characteristics for individuals with pediatric-onset SCI living in rural areas vs. urban areas.

Characteristics	Urban		Rural		Significance test
	(*n* = 388)		(*n* = 102)		
	*M* (SD)	Median	*M* (SD)	Median	
Age	34.37 (7.36)	33.42	33.82 (8.02)	32.69	*t* = .537, *p* = .592
Age at injury	14.13 (4.45)	15.63	14.41 (4.51)	15.65	*t* = −.556, *p* = .579
Injury duration	20.19 (8.33)	19.21	19.41 (9.19)	17.58	*t* = .827, *p* = .408
	***n*** (%)	***n*** (%)	
Sex					*χ*^2^ (1) = 3.64, *p* = .056
Male	234 (60.3%)	72 (70.6%)	
Female	154 (39.7%)	30 (29.4%)	
Race					*χ*^2^ (1) = 2.82, *p* = .093
Caucasian	325 (84.4%)	91 (91%)	
Other	60 (15.6%)	9 (9%)	
Injury level					*χ*^2^ (1) = .005, *p* = .944
Paraplegia	180 (46.4%)	46 (46%)	
Tetraplegia	208 (53.6%)	54 (54%)	
Complete/incomplete					*χ*^2^ (1) = .693, *p* = .405
Complete	262 (69.7%)	64 (65.3%)	
Incomplete	114 (30.3%)	34 (34.7%)	
AIS injury severity					*χ*^2^ (3) = 3.59, *p* = .309
A	49 (12.8%)	18 (18.2%)	
B	137 (35.9%)	27 (27.3%)	
C	160 (41.9%)	43 (43.4%)	
D	36 (9.4%)	11 (11.1%)	
Etiology					*χ*^2^ (4) = 4.72, *p* = .318
Vehicular/pedestrian	184 (47.9%)	60 (58.8%)	
Violence	37 (9.6%)	6 (5.9%)	
Fall/flying object	24 (6.3%)	7 (6.9%)	
Sports	97 (25.3%)	21 (20.6%)	
Medical/surgical	42 (10.9%)	8 (7.8%)	

**Table 2:** Comparison of structural and healthcare characteristics for individuals with pediatric-onset SCI living in rural areas vs. urban areas.

Characteristics	Urban		Rural		Significance test
	(*n* = 388)		(*n* = 102)		
	*M* (SD)	Median	*M* (SD)	Median	
Personal income	39906.52 (71,386.75)	21800.00	20825.20 (19854.66)	10800.00	*U* = 12412.50, ***p* = <.001**
Household income	58562.43 (74,894.43)	41000.00	38864.50 (32667.16)	30000.00	*t* = 5.59, ***p* = .023**
Paid assistance hours for personal care	1.66 (3.09)	0.00	1.18 (2.61)	0.00	*U* = 17456.00, *p* = .053
Unpaid assistance hours for personal care	0.74 (1.92)	0.00	1.24 (2.85)	0.00	*U *= 21375.50, ***p* = .035**
Out of pocket medical expenses	4017.37 (11,177.91)	1000.00	2092.96 (3431.89)	600.00	*U *= 11967.00, *p* = .236
Hours worked per week	38.92 (14.29)	40.00	42.28 (17.05)	42.50	*t* = 3.69, ***p* = .002**
	***n*** (%)	***n*** (%)	
Education					*χ*^2^ (1) = 17.71, ***p* = <.001**
College degree or higher	185 (47.7%)	25 (24.5%)	
No college degree	203 (52.3%)	77 (75.5%)	
Work status					*χ*^2^ (1) = 14.78, ***p* < .001**
Employed	201 (57.3%)	32 (34.8%)	
Unemployed	150 (42.7%)	60 (65.2%)	
Insurance					*χ*^2^ (1) = 8.44, ***p* = .015**
Private insurance	188 (50.1%)	36 (35.6%)	
Public insurance or none	187 (49.9%)	65 (65.4%)	

Bold values denote statistical significance at the *p* < 0.05 level.

After controlling for race, injury level, and age at injury, a series of logistic regressions revealed significant differences between individuals residing in rural vs. urban areas, which are presented in Table 3. In terms of secondary health conditions, those residing in rural regions more frequently experienced a pressure injury (*p* = .007) and greater frequency of UTIs (*p* = .009) and bowel incontinence (*p *= .027) within the previous year. Moreover, this group was significantly more likely to report being hospitalized for some medical purpose (i.e., non-psychiatric hospitalization) within the past year (*p* = .032). Individuals residing in rural areas were more likely to report sleep challenges (*p* = .024) and lower perceived physical health as well (*p* = .035). There were no differences in reported bladder incontinence or pain characteristics. The two groups did not appear to differ in terms of various psychosocial variables, including depressive symptoms, anxiety, general level of happiness, satisfaction with life, and perceived mental health. In terms of community integration as measured by the CHART, there were no differences reported in terms of physical, cognitive, or economic independence; however, rural-living participants were significantly less likely to be independent and participatory in terms of mobility (*p* = .002), occupation (*p* = .014), and social integration (*p *< .001).

**Table 3:** Adjusted odds ratios for rural (vs. urban) living predicting secondary health conditions, psychosocial functioning, and community integration in individuals with pediatric-onset SCI.

Outcome variable	Adjusted OR (95% CI)	*p* value
**Secondary health conditions**
Pressure injury	1.88 (1.19–2.97)	**.** **007**
Urinary tract infection (UTI)	0.94 (0.94–2.64)	.087
Number of UTIs	1.08 (1.02–1.15)	**.** **009**
Hospitalizations	1.68 (1.05–2.71)	**.** **032**
Bladder incontinence	0.81 (0.67–1.67)	.805
Bowel incontinence	1.91 (1.08–3.39)	**.** **027**
Current level of pain	1.07 (0.94–1.21)	.310
Frequency of pain	1.11 (0.96–1.30)	.168
Sleep difficulties	1.09 (1.01–1.18)	**.** **024**
**Psychosocial outcomes**
Depressive symptoms	1.03 (0.97–1.08)	.378
Anxiety	1.00 (0.95–1.05)	.904
Satisfaction with life	1.00 (0.97–1.02)	.676
General happiness	1.15 (0.86–1.52)	.353
Perceived physical health	0.98 (0.95–1.00)	**.** **036**
Perceived mental health	1.01 (0.99–1.04)	.353
**Community integration**
Physical independence	1.00 (0.97–1.01)	.486
Cognitive independence	0.98 (0.95–1.00)	.060
Mobility	0.98 (0.97–0.99)	**.** **002**
Occupation	0.99 (0.98–1.00)	**.** **014**
Social integration	0.97 (0.96–0.99)	**<** **.** **001**
Economic self-sufficiency	0.99 (0.99–1.00)	.074

Bold values denote statistical significance at the *p* < 0.05 level.

## 4. Discussion

To the authors’ knowledge, no study has examined the impact of geographical location on the long-term health outcomes of patients with pediatric-onset SCI. Within the cohort involved in the current study, 20% of participants lived in rural areas, which is consistent with the national average of 19% (36). A previous study of veterans with SCIs showed a higher proportion living in rural areas (39.2%) (37) indicating that the present sample may be more representative of the general U.S. population. The results of the current study indicated higher structural and healthcare concerns and more secondary health conditions for individuals with pediatric-onset SCI residing in rural regions vs. individuals residing in urban areas. Specifically, those residing in rural environments were more likely to have lower education, lower income, longer work hours, reduced access to private health insurance, greater need for unpaid personal care assistance, and be unemployed. They also endorsed other factors affecting healthcare usage, including higher concerns regarding mobility, occupation, and social engagement. In terms of health outcomes, rural-living individuals were at greater risk for pressure injuries, bowel incontinence, increased number of UTIs, and sleep difficulties than individuals living in urban regions.

As predicted, residing in a rural vs. urban area appeared to be associated with a variety of structural, financial, and healthcare access characteristics for individuals with pediatric-onset SCI. With respect to education, the participants living in urban regions were more educated, having a higher attainment of college degree than the national average (34%) (38). U.S. Census Bureau, 2018). It is possible that having an SCI early in life predisposes individuals to seeking further education to improve future employment opportunities. It may also mean that these individuals have the potential to be more adaptable to their SCI than those that experience injury later in life. Future research should examine specific educational and vocational differences between pediatric-onset and adult-onset SCI groups as well as to test the mechanisms that may explain these differences. In the current sample, there was indeed a notable distinction between the geographical groups, with rural-living individuals reporting significantly lower levels of educational attainment, potentially predisposing these individuals to reduced levels of employment and reduced income. This disparity highlights that individuals with pediatric-onset SCI living in rural environments may be at increased risk of not keeping pace with peers as they enter adulthood.

In addition to educational attainment, the urban-living participants in this study were more likely to be employed, have higher incomes, and work fewer hours, which would be consistent with prior studies showing increased opportunities in urban and higher socioeconomic status areas (39). Rural-living individuals also had lower personal yearly incomes, with an overall average difference of over $19,000, which is notably more than the national difference of $15,779 observed in the general population (36). The relationship between income and urbanicity remains complex as individuals may relocate to urban environments for higher education and for work. Individuals may also choose to live in rural areas when employed or underemployed where cost of living is more affordable. There may also be variations in family makeup (e.g., living with parent, caregiver, spouse vs. living alone) between rural and urban and future studies should examine the influence of this factor on income and educational attainment. However, it appears that those living in urban environments may be at overall lower risk given the financial burden that having a SCI confers in addition to the positive influence of employment and income on life expectancy and satisfaction (3). Urban areas also help by providing independent living, mobility, and functional independence, which predict employment (40).

The two groups in our sample also significantly differed in terms of health insurance coverage, with rurality being linked with having no health insurance or reliance on government-subsidized insurance. This discrepancy, which could be due to disparities in employment and decreased income as well as distance to care, is likely consequential given the high economic burden of healthcare following SCI. Additionally, individuals with Medicaid/Medicare may not receive adequate care given limited access to SCI specialists and a higher rate of utilizing non-specialty services, such as emergency rooms, where knowledge of SCI is lower (41). Indeed, having subsidized health insurance has been linked with higher rates of hospitalizations in the pediatric-onset SCI population (10). Having limited healthcare coverage, combined with reduced economic resources including preventive care, may place rural-living individuals at further risk of developing secondary health complications. Encouragingly, there were no significant differences in out-of-pocket medical expenses or hours of paid home assistance across the groups. However, this finding may be explained by the lower cost of living in rural regions, the limited income of the rural-living individuals, or lack of area health services found in these regions (2).

With respect to CHART scores, individuals living in rural regions differed significantly on three dimensions of participation and independence in the community, including mobility, occupation, and social integration. These finding indicate that those in rural environments are at increased risk for reduced community integration even after controlling for race, injury level, and age at injury. An individual with SCI living in rural environments may experience reduced capacity to navigate their area effectively and efficiently with limited access to means of transportation options, physical and architectural barriers, and distance from community assets. In addition, these same barriers of greater distance to accessible social and recreational opportunities may also influence an individual’s occupational and social integration (39).

Contrary to our hypotheses, no differences emerged between the rural and urban groups in terms of anxiety and depressive symptoms, happiness, life satisfaction, and perceived quality of life. It is possible that experiencing a SCI in youth may be protective in our sample when compared with individuals with adult-onset SCI. Indeed, previous studies have demonstrated that individuals who experience SCI at older ages are at elevated risk for depression, anxiety, and lower satisfaction with life (34, 35). The similarities in patient psychosocial outcomes in those living in rural and urban settings may indicate that patients can achieve similar outcomes regardless of residence. Nevertheless, given the inequities identified in terms of service utilization and access, further studies are recommended to replicate the results of the current investigation and to potentially evaluate needs for mental health delivery services to this vulnerable population.

The confluence of the aforementioned structural, financial, and healthcare access risk factors present in rural environments may have also contributed to the finding that rural-living individuals with pediatric-onset SCI reported higher incidence of long-term secondary health conditions, including pressure injuries, bowel incontinence, number of reported UTIs, and sleep difficulties. Rural-living individuals, who as noted previously report less comprehensive health insurance coverage, lower incomes, lower education, and lower levels of employment, may have inadequate access to quality healthcare which in turn leads to difficulty accessing various forms of preventative healthcare, which could mitigate vulnerability to secondary health complications. For example, Goodrich and colleagues (2) noted that rehabilitative services such as physical and occupational therapy—highly valued by participants in preventing pressure injuries—may be more difficult to access in rural settings.

Access to services is an integral component in healthcare in rural regions, and though some barriers to care are intractable, such as long transportation distances, others are modifiable, such as the supply of providers (42) as well as characteristics of the health delivery system. Reducing access disparities could be partially accomplished by providing more telehealth options for rural residents to augment access to specialized healthcare services. Other telehealth possibilities may involve the provision of health education related to preventative self-management or peer support [see McIntyre (43) and Barker (44) for reference]. Additionally, behavioral interventions designed to promote health education and achievement of health-related goals, such as problem-solving education (45) or health coaching (46, 47) may be provided in a telehealth context to individuals with SCI living in rural environments. Moreover, the provision of outreach services and small outpatient clinics may be instrumental in preventing long-term health complications (48, 49). There is also evidence that home-based interventions are efficacious in improving lifestyle behaviors and early detection among individuals with chronic health conditions (19). Such programs may be effectively applied for individuals living with SCI in rural regions of the country.

Although the current study provides valuable information related to the disparities observed between rural and urban residing individuals with pediatric-onset SCI, there are limitations to this study that are worth acknowledging. Generalizability of the current study is limited as participants were predominantly Caucasian and male, although this reflects data from the general U.S. SCI Model Systems (50). Nevertheless, the present study benefited from a large (*N* = 471) and geographically diverse sample as well as long-term follow-up. Another limitation involves using zip codes as a measurement of rurality, which may be a crude approximation of the actual geographical type of an individual’s community or the assets attainable to an individual near their home. The use of more accurate measurements of rurality, like the index of relative rurality (51), have been proposed; however, the ProximityOne database lacks the necessary input variables to employ this method. Data regarding community assets, distance traveled to seek medical care, and specific transportation needs would be useful predictors to examine. Future studies should integrate more refined measures of rurality to determine if the health disparities identified by this study remain true.

Another important limitation to acknowledge relates to the analytic approach employed in the current study. Given the high number of outcomes included in this study, numerous statistical models were performed to compare the differences between the two groups (rural and urban) in the sample, inflating the likelihood of Type I error. However, as there is not an established relationship between geographical location and the outcome variables of interest and no existing studies examining these relationships within the pediatric-onset SCI population, the current study is more exploratory in nature. Thus, it was determined that statistical corrections for multiplicity were not conducted owing to the potential rise of Type II error and the dismissal of potentially important disparities identified between these two groups [see Feise (52) for reference]. Nevertheless, given the multiple tests conducted in this study and the associated elevated possibility of familywise Type I error, the reader is encouraged to interpret significant findings with appropriate caution.

This study offers evidence that rural living in adults with pediatric-onset SCI may confer risk and potentially predict diminished educational attainment, unemployment, lack of healthcare resources, preventable hospitalization, and poorer long-term health outcomes compared to individuals living in urban environments. Financial and structural challenges to obtaining appropriate healthcare and rehabilitative services may explain the increased incidence of secondary health conditions in this population. These findings warrant continued research to further understand how community and geographical location in pediatric-onset SCI may related to adulthood outcomes and suggest that youth with SCI who reside in rural environments may require increased access to services to promote and maintain long-term health and well-being.

## Data Availability

The datasets presented in this article are not readily available because Shriners Children's Research Programs is not positioned to permit the external sharing of research datasets at this time. Requests to access the datasets should be directed to KZ, Ph.D., Chief of Psychology at Shriners Children's Chicago at kzebracki@shrinenet.org.
